# Supplementary material for: De novo atomic protein structure modeling for cryoEM density maps using 3D transformer and HMM
Source: Nat Commun. 2024 Jun 29;15:5511. doi: 10.1038/s41467-024-49647-6 (PMC11217428; doi:10.1038/s41467-024-49647-6)
Supplement: Supplementary file 1 — Supplementary Information [file 41467_2024_49647_MOESM1_ESM.pdf]

De novo atomic protein structure modeling for CryoEM density maps using 3D transformer and HMM

Nabin Giri[1,2] and Jianlin Cheng[1,2*]

[1]Department of Electrical Engineering and Computer Science, University of Missouri, Columbia, 65211, Missouri, USA.
[2]Roy Blunt NextGen Precision Health, University of Missouri, Columbia, 65211, Missouri, USA.

*Corresponding author(s). E-mail(s): chengji@missouri.edu;
Contributing authors: ngzvh@missouri.edu;

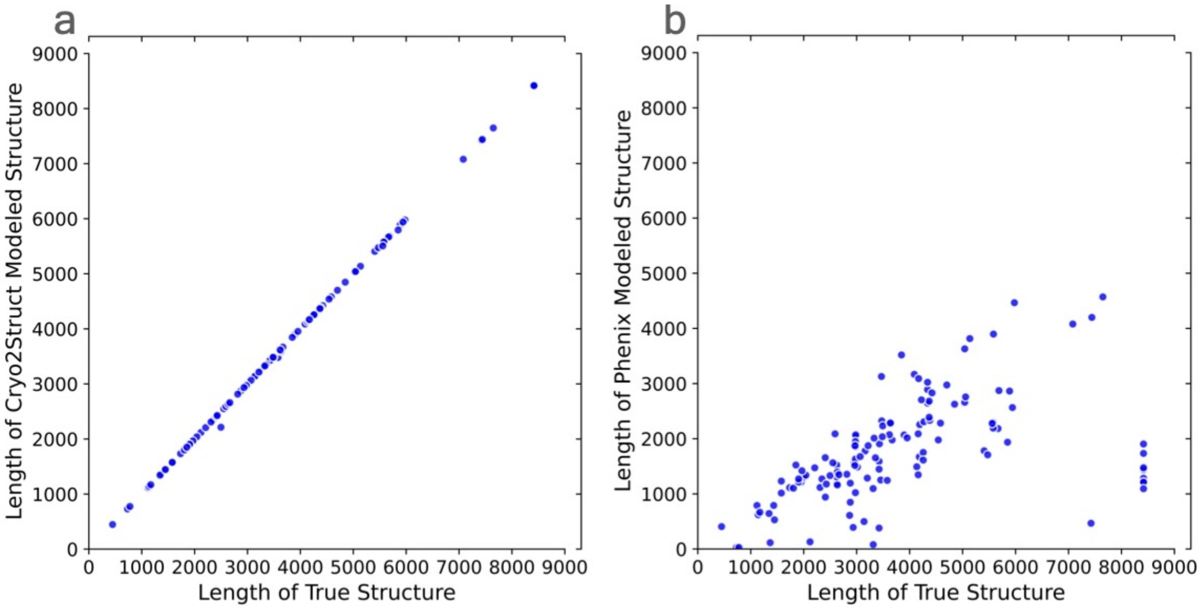

Fig. S1 Length of structural models built by Cryo2Struct and Phenix versus (VS) length of the true structures in the standard test dataset. (**a**) Cryo2Struct models VS true structures. (**b**) Phenix models VS true structures. Source data are provided as a Source Data file.

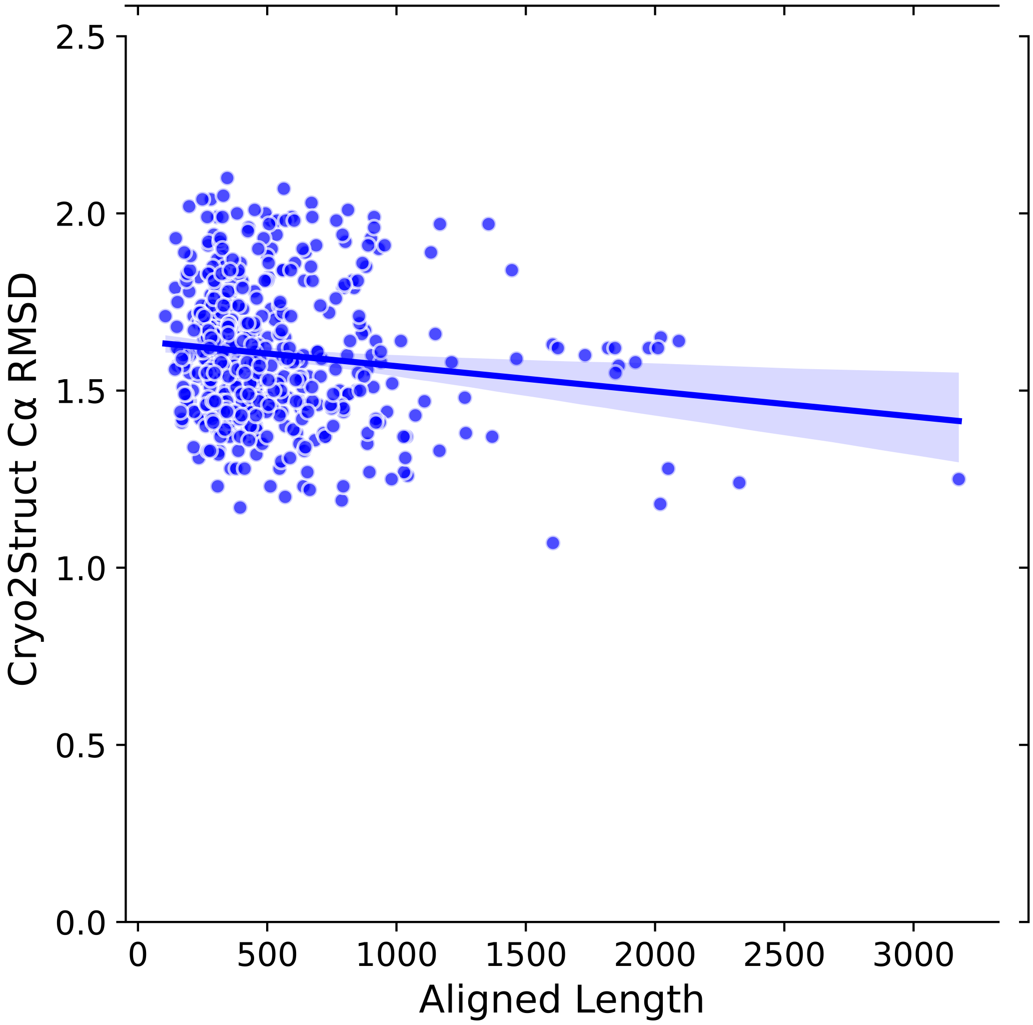

Fig. S2 RMSD versus the length of the aligned regions of the atomic models built for 500 test cryo-EM maps. The models were aligned with the true structures by US-align. The solid line depicts linear regression line, and the colored area represents a 95% confidence interval. The regression equation: $y = -0.0001x + 1.6401$; the correlation: -0.134. The average RMSD of the models is 1.60 Å. The average aligned length is 532.51 where as the average length of true structure is 1837.43. Cryo2Struct models have about 29% aligned length. Source data are provided as a Source Data file.

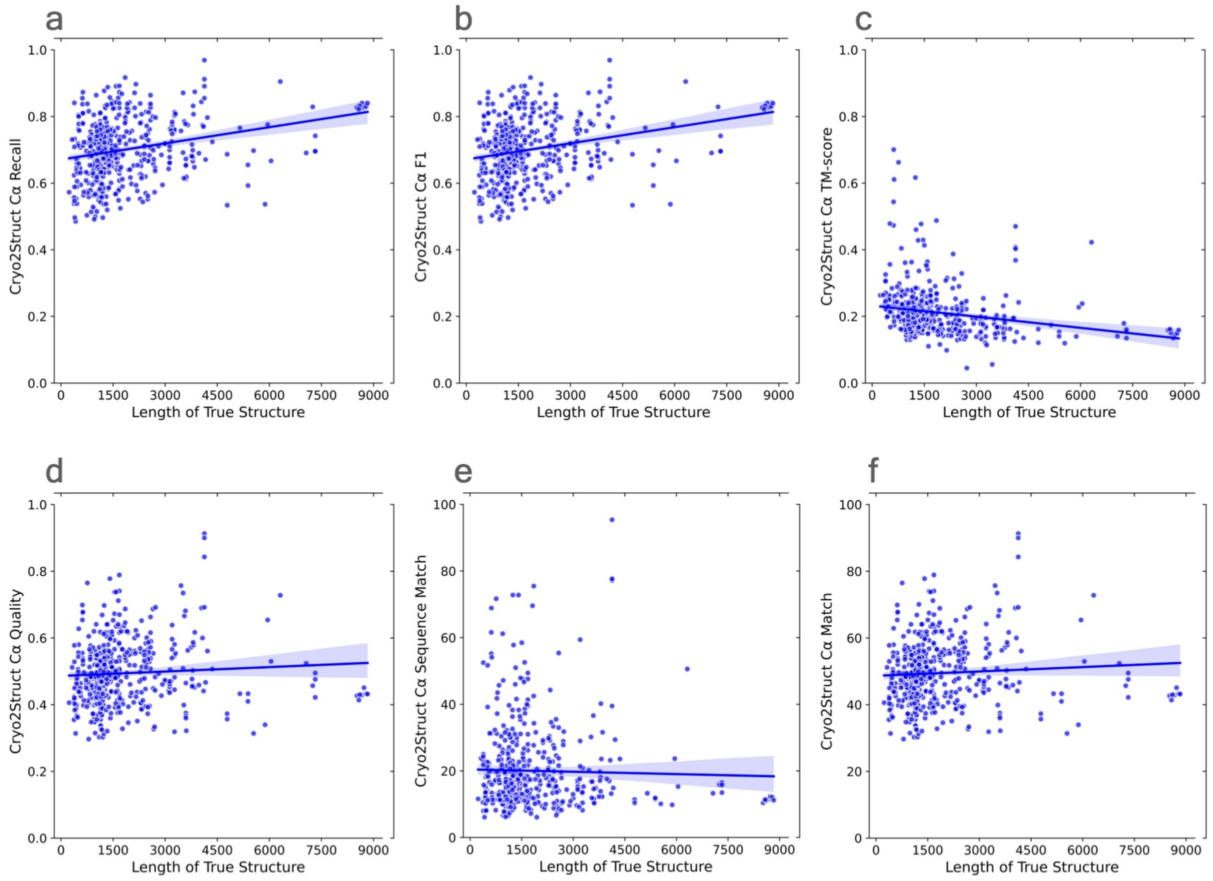

Fig. S3 The quality scores of atomic models built for the 500 cryo-EM maps in the new test dataset versus (VS) the length of the true structures. The solid lines depicts linear regression lines, and the colored area represents a 95% confidence interval. (**a**) The Cα recall VS length of true structure; the regression equation: $0.0000x + 0.6712$; Pearson's correlation: 0.259. (**b**) The F1 score VS length of true structure; the regression equation: $0.0000x + 0.6714$; the correlation: 0.258. (**c**) The normalized TM-score VS length of true structure; the regression equation: $-0.0000x + 0.2328$; the correlation: -0.214. (**d**) The Cα quality score VS length of true structure; the regression equation: $0.0000x + 0.4863$; the correlation: 0.066. (**e**) The Cα sequence match score VS length of true structure; the regression equation: $-0.0002x + 20.4579$; the correlation: -0.025. (**f**) The Cα match score VS length of true structure; the regression equation: $0.0004x + 48.6615$; the correlation: 0.065. Source data are provided as a Source Data file.

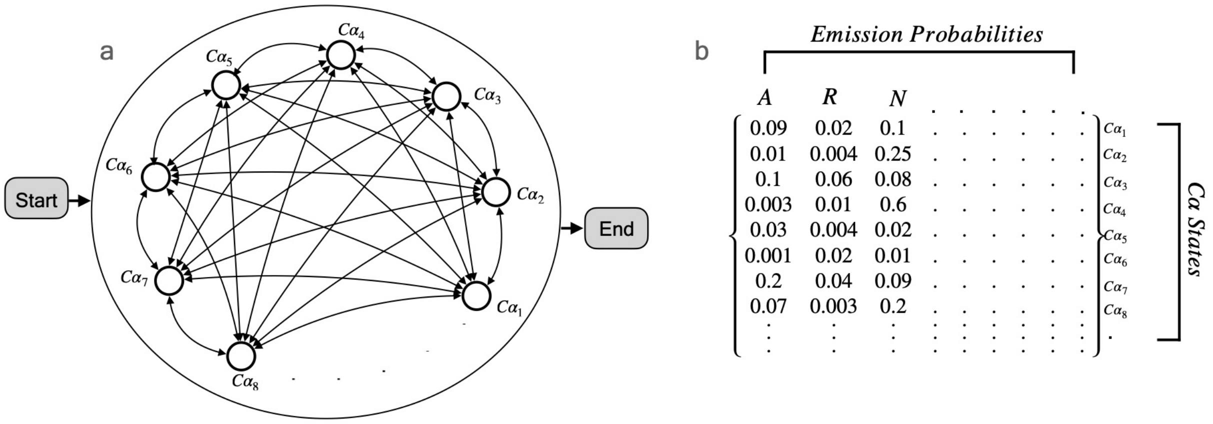

Fig. S4 A Hidden Markov Model (HMM) used for aligning protein sequences with predicted Cα atoms (voxels) to generate protein backbone traces. (a) The states of the fully connected HMM. A hidden path can start from or end at any Cα state. It is worth noting that there is no gap state in the HMM and therefore every amino acid in a protein sequence can be aligned to one Cα atom. **(b)** The emission probabilities of the hidden Cα states are the normalized geometric mean of the predicted amino acid type probability and the background (prior) probability for 20 amino acids in the nature, referred to by their abbreviation.

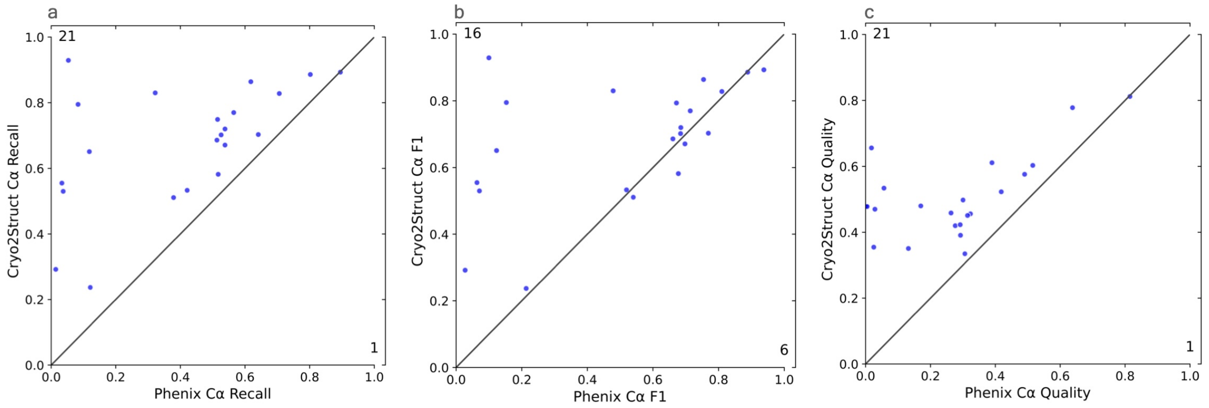

Fig. S5 The comparative analysis of atomic models built for 22 cryo-EM density maps in the redundancy-reduced standard test dataset by Cryo2Struct and Phenix in terms of recall, F1 score, and quality score of Cα atoms. The proteins of the density maps have $\leq 25\%$ sequence identity with the protein in the training and validation datasets. In the panel of each evaluation metric, the score of the model built by Cryo2Struct for each map is plotted against that by Phenix for the same map. A dot above the 45 degree line indicates that Cryo2Struct has higher score than Phenix for the map. The number in the top-left corner represents the total number of maps on which Cryo2Struct has higher scores, while the number in the bottom-right corner denotes the total number of maps on which Phenix has higher scores. **(a)** The recall of Cα. **(b)** The F1 score of Cα. **(c)** The Cα quality score. Source data are provided as a Source Data file.

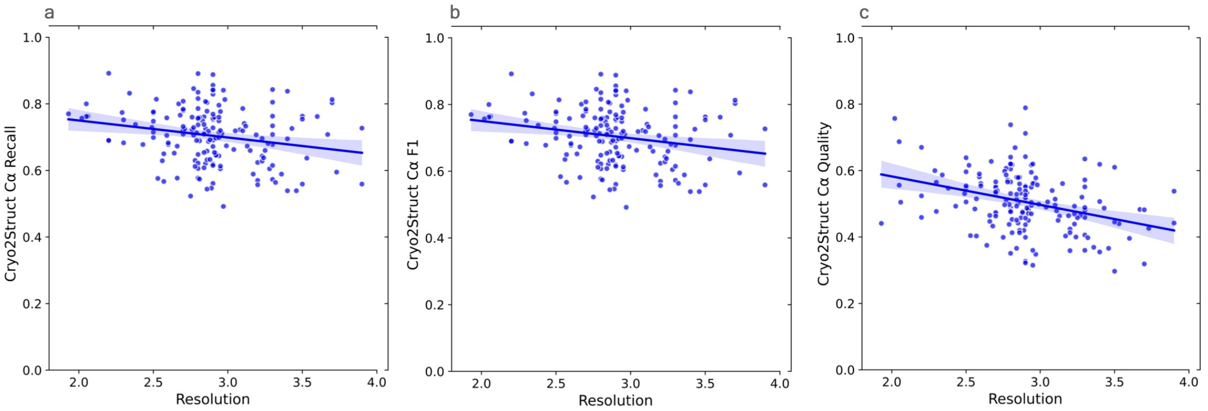

Fig. S6 The scores of atomic models built by Cryo2Struct for 169 test cryo-EM maps in the redundancy-reduced new test dataset plotted against the resolution of the maps. The proteins of the density maps have $\leq$ 25% sequence identity with the protein in the training and vaidation datasets. The solid lines depict linear regression lines, and the colored area represents a 95% confidence interval. (**a**) The Cα recall versus resolution; the regression equation: $-0.0511x + 0.8521$; Pearson's correlation: -0.217. (**b**) The F1 score versus resolution; the regression equation: $-0.0515x + 0.8536$; the correlation: -0.219. (**c**) The quality score versus resolution; the regression equation: $-0.0856x + 0.7537$; the correlation: -0.344. Source data are provided as a Source Data file.

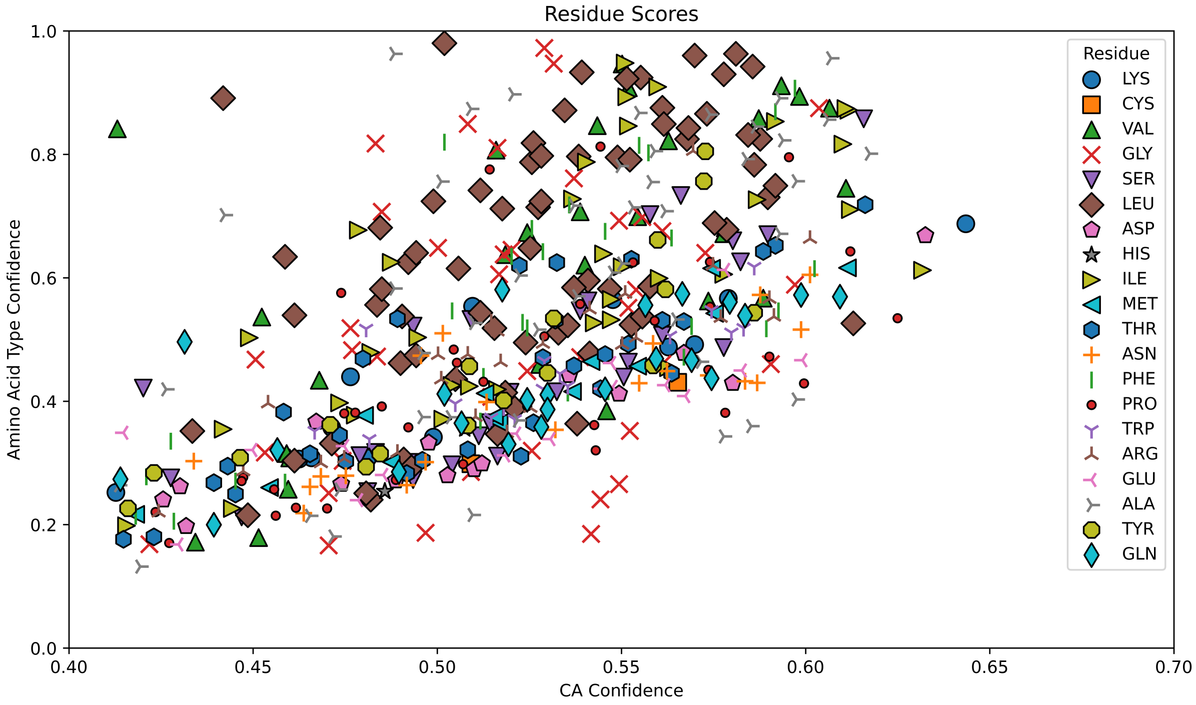

Fig. S7 The residue-wise confidence scores provided by Cryo2Struct pertaining to the modeled structure for the cryo-EM density map with the EMD ID: 15789 (PDB ID: 8B0N, released on 2023-07-12, and resolution of 2.67 Å). The x-axis represents the confidence scores of predicted Cα atoms. The y-axis denotes the confidence scores associated with the amino acid types for the Cα atoms. The different shapes in the plot denote different amino acid types. The average Cα confidence score is 0.53, while the average confidence score for amino acid types is 0.511. The total number of modeled residues is 510. There is a clear positive correlation between the two kinds of confidence scores.

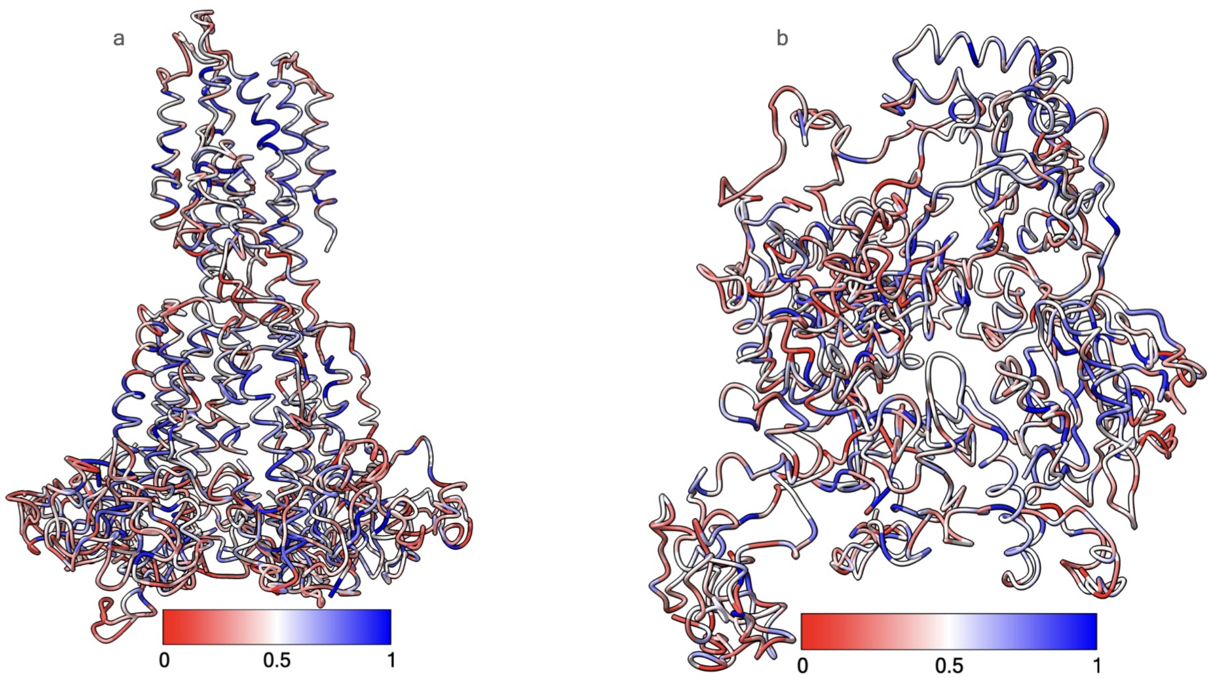

Fig. S8 The residue-wise amino acid type confidence scores mapped to the modeled structure and visualized using a color spectrum. **(a)** Cryo2Struct modeled structure for the cryo-EM density map with the EMD ID: 41624 (PDB ID: 8TUL, released on 2023-09-13, resolution of 2.8 Å). **(b)** Cryo2Struct modeled structure for the cryo-EM density map with the EMD ID: 34402 (PDB ID: 8GZR, released on 2023-08-02, and resolution of 2.8 Å). Both **(a)** and **(b)** have less than 25% sequence identity with the proteins in the dataset used to train the deep learning model.

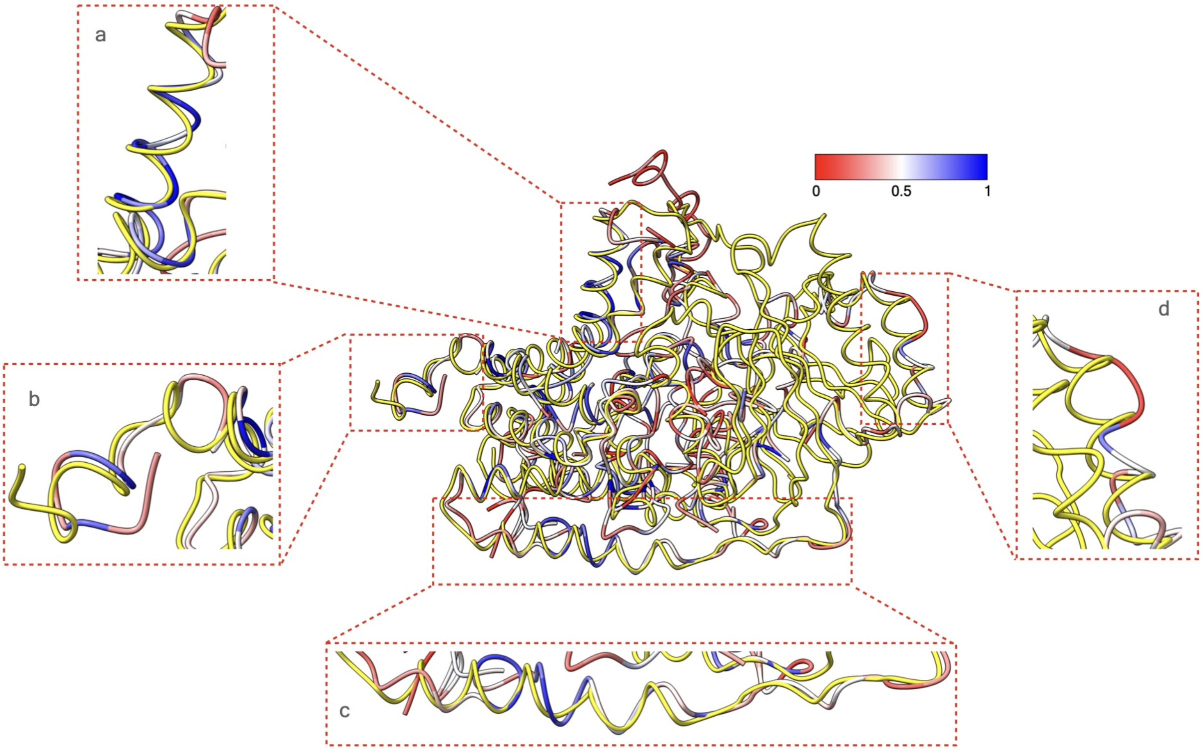

Fig. S9 An in-depth analysis of residue-wise amino acid type confidence scores, mapped onto the Cryo2Struct modeled structure and visualized through a color spectrum, for EMD ID: 15789. The modeled structure has less than 25% sequence identity with the proteins in the dataset used to train the deep learning model. The known PDB structure (PDB ID: 8B0N) is depicted in yellow color. **(a)** A segment of the well modeled region with high confidence scores, particularly within helical motifs. **(b)** A mixed region of different quality exhibiting different confidence scores. **(c)** An extended segment of the modeled structure with varying confidence levels, ranging from high to low, compared to the known structure. **(d)** Low confidence scores are observed in the regions where the modeled helix substantially deviates from the known PDB structure, indicating uncertainty.

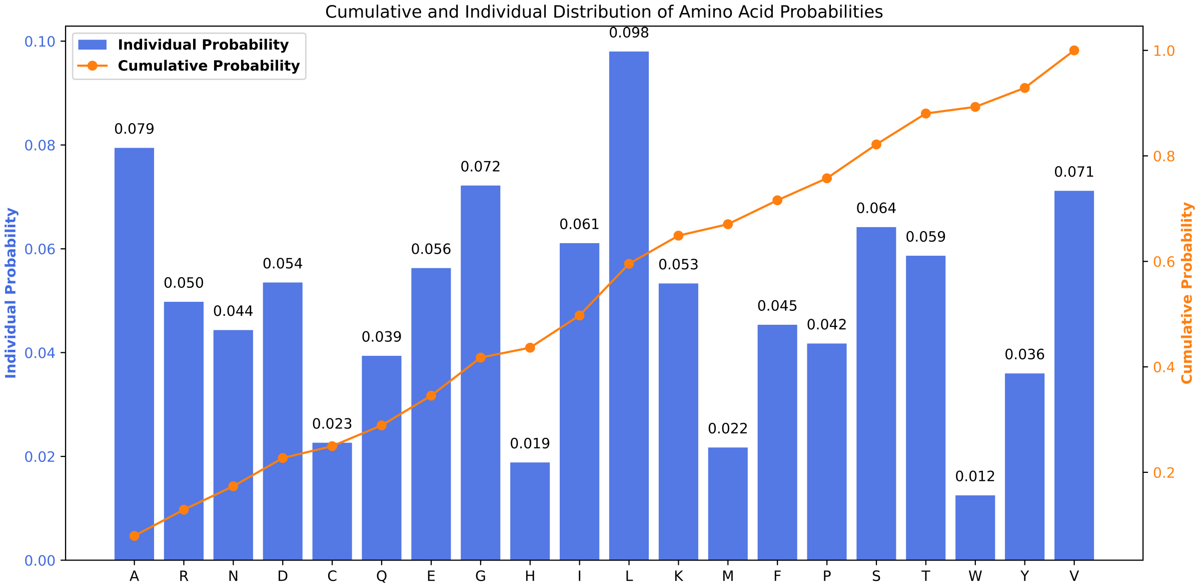

Fig. S10 The bar plot visualizing the individual probability (frequency) of each amino acid type in the training dataset. Complementing this, the cumulative distribution function (CDF) is presented on the secondary y-axis (orange), elucidating the probability distribution of amino acid types in the dataset, summing up to 1. This visualization offers a comprehensive depiction of prior amino acid probabilities, which are combined with the probabilities of amino acid types predicted by Cryo2Struct to construct the emission probabilities of amino acid types in the HMM.
