# Supplementary material for: De novo atomic protein structure modeling for cryoEM density maps using 3D transformer and HMM
Source: Nat Commun. 2024 Jun 29;15:5511. doi: 10.1038/s41467-024-49647-6 (PMC11217428; doi:10.1038/s41467-024-49647-6)
Supplement: Supplementary file 2 — Reporting Summary [file 41467_2024_49647_MOESM2_ESM.pdf]

nature portfolio

Corresponding author(s): Jianlin Cheng

Last updated by author(s): Jun 6, 2024

Reporting Summary

Nature Portfolio wishes to improve the reproducibility of the work that we publish. This form provides structure for consistency and transparency in reporting. For further information on Nature Portfolio policies, see our Editorial Policies and the Editorial Policy Checklist.

Statistics

For all statistical analyses, confirm that the following items are present in the figure legend, table legend, main text, or Methods section.

n/a	Confirmed	
☐	☒	The exact sample size (*n*) for each experimental group/condition, given as a discrete number and unit of measurement
☐	☒	A statement on whether measurements were taken from distinct samples or whether the same sample was measured repeatedly
☐	☒	The statistical test(s) used AND whether they are one- or two-sided<br>*Only common tests should be described solely by name; describe more complex techniques in the Methods section.*
☒	☐	A description of all covariates tested
☒	☐	A description of any assumptions or corrections, such as tests of normality and adjustment for multiple comparisons
☐	☒	A full description of the statistical parameters including central tendency (e.g. means) or other basic estimates (e.g. regression coefficient) AND variation (e.g. standard deviation) or associated estimates of uncertainty (e.g. confidence intervals)
☒	☐	For null hypothesis testing, the test statistic (e.g. *F*, *t*, *r*) with confidence intervals, effect sizes, degrees of freedom and *P* value noted<br>*Give P values as exact values whenever suitable.*
☐	☒	For Bayesian analysis, information on the choice of priors and Markov chain Monte Carlo settings
☒	☐	For hierarchical and complex designs, identification of the appropriate level for tests and full reporting of outcomes
☐	☒	Estimates of effect sizes (e.g. Cohen's *d*, Pearson's *r*), indicating how they were calculated

Our web collection on statistics for biologists contains articles on many of the points above.

Software and code

Policy information about availability of computer code

Data collection	We used the open-sourced Cryo2StructData dataset for training and validating the Cryo2Struct method. The standard test dataset used to compare Phenix and Cryo2Struct is also from the test dataset of Cryo2StructData. We downloaded the standard Phenix-modeled atomic structure from the Phenix website (https://phenix-online.org/phenix_data/terwilliger/map_to_model_2018/). The modeled structures for Cryo2Struct, both for the standard (128 test maps) and for the new cryo-EM density maps 500 test maps), are released in the Cryo2Struct Dataverse, available here: https://doi.org/10.7910/DVN/GQCTTD. We followed the same preprocessing steps carried out by the Cryo2StructData authors while preparing the new 500 test dataset. A detailed description of the preprocessing of Cryo2StructData is available here (doi: https://doi.org/10.1101/2023.06.14.545024). The Cryo2StructData dataset is available here: https://doi.org/10.7910/DVN/FCDG0W. Below, we provide a brief overview and the software package/version used.<br><br>We downloaded the cryo-EM density maps from the Electron Microscopy Data Bank (EMDB) using a bash script. The corresponding PDB (protein structures) were downloaded from the Protein Data Bank (PDB) using a Python script. Both the bash scripts and Python script to download maps and atomic structures are available in the Cryo2StructData GitHub repository. The label generation process, as described in the Cryo2StructData paper, is also available in the Cryo2StructData GitHub repository. We used Python 3.9.18 installed in a conda environment. The label generation process makes use of two Python open-source packages named mrcfile (version 1.4.3) and biopython (version 1.81). To model atomic structure in cryo-EM density maps, Cryo2Struct first resamples the cryo-EM density map to a 1 Angstrom voxel size. The resampling step is carried out using UCSF ChimeraX 1.4-1 on a AlmaLinux 8.9 system in non-GUI mode. After resampling, we carried out normalization of the cryo-EM density maps using a Python program available in the Cryo2Struct GitHub repository.<br><br>Cryo2StructData GitHub repository: https://github.com/BioinfoMachineLearning/cryo2struct.git<br>Cryo2Struct GitHub repository: https://github.com/jianlin-cheng/Cryo2Struct.git<br>Cryo2Struct Source Code DOI : https://doi.org/10.5281/zenodo.11492584
Data analysis	We utilized Phenix-1.20.1-4487, installed locally on a AlmaLinux 8.9 system, to compute the carbon-alpha match score, carbon-alpha sequence match score, and carbon-alpha quality scores. Additionally, we employed US-align (Version 20230609), installed locally on a AlmaLinux 8.9 system, to compute the global normalized TM-score, aligned carbon-alpha length, and total modeled carbon-alpha atoms in the known structure. The regression line with a degree of 1 was produced using the seaborn package (version 0.12.2), and the Pearson correlation coefficient was computed using the pandas package (version 1.5.3).<br><br>The structural models built for the 128 test cryo-EM maps in the test data by Cryo2Struct and Phenix (for Phenix, the structures were downloaded from: https://phenix-online.org/phenix_data/terwilliger/map_to_model_2018/) were compared with the true structures in the Protein Data Bank (PDB) to evaluate their quality. Similarly, the structural models built for the new 500 test cryo-EM maps by Cryo2Struct were also compared with the true structures in the Protein Data Bank (PDB) to evaluate their quality.

For manuscripts utilizing custom algorithms or software that are central to the research but not yet described in published literature, software must be made available to editors and reviewers. We strongly encourage code deposition in a community repository (e.g. GitHub). See the Nature Portfolio guidelines for submitting code & software for further information.

Data

Policy information about availability of data

All manuscripts must include a data availability statement. This statement should provide the following information, where applicable:

- Accession codes, unique identifiers, or web links for publicly available datasets
- A description of any restrictions on data availability
- For clinical datasets or third party data, please ensure that the statement adheres to our policy

The training and validation dataset used for Cryo2Struct are available in the Cryo2StructData Dataverse, accessible here: https://doi.org/10.7910/DVN/FCDG0W. The modeled atomic structures by Phenix (downloaded from the Phenix website: https://phenix-online.org/phenix_data/terwilliger/map_to_model_2018/) and Cryo2Struct, along with the known atomic structures, are available in the Cryo2Struct Dataverse, accessible here: https://doi.org/10.7910/DVN/GQCTTD

Research involving human participants, their data, or biological material

Policy information about studies with human participants or human data. See also policy information about sex, gender (identity/presentation), and sexual orientation and race, ethnicity and racism.

Reporting on sex and gender	*Use the terms sex (biological attribute) and gender (shaped by social and cultural circumstances) carefully in order to avoid confusing both terms. Indicate if findings apply to only one sex or gender; describe whether sex and gender were considered in study design; whether sex and/or gender was determined based on self-reporting or assigned and methods used.*<br>*Provide in the source data disaggregated sex and gender data, where this information has been collected, and if consent has been obtained for sharing of individual-level data; provide overall numbers in this Reporting Summary. Please state if this information has not been collected.*<br>*Report sex- and gender-based analyses where performed, justify reasons for lack of sex- and gender-based analysis.*
Reporting on race, ethnicity, or other socially relevant groupings	*Please specify the socially constructed or socially relevant categorization variable(s) used in your manuscript and explain why they were used. Please note that such variables should not be used as proxies for other socially constructed/relevant variables (for example, race or ethnicity should not be used as a proxy for socioeconomic status).*<br>*Provide clear definitions of the relevant terms used, how they were provided (by the participants/respondents, the researchers, or third parties), and the method(s) used to classify people into the different categories (e.g. self-report, census or*

	administrative data, social media data, etc.) *Please provide details about how you controlled for confounding variables in your analyses.*
Population characteristics	*Describe the covariate-relevant population characteristics of the human research participants (e.g. age, genotypic information, past and current diagnosis and treatment categories). If you filled out the behavioural & social sciences study design questions and have nothing to add here, write "See above."*
Recruitment	*Describe how participants were recruited. Outline any potential self-selection bias or other biases that may be present and how these are likely to impact results.*
Ethics oversight	*Identify the organization(s) that approved the study protocol.*

Note that full information on the approval of the study protocol must also be provided in the manuscript.

Field-specific reporting

Please select the one below that is the best fit for your research. If you are not sure, read the appropriate sections before making your selection.

[x] Life sciences [ ] Behavioural & social sciences [ ] Ecological, evolutionary & environmental sciences

For a reference copy of the document with all sections, see nature.com/documents/nr-reporting-summary-flat.pdf

Life sciences study design

All studies must disclose on these points even when the disclosure is negative.

Sample size	First, the entire Electron Microscopy Data Bank (EMDB) was queried for single-particle cryo-EM density maps with resolutions between 0 and 4 Angstrom (high and medium-resolution cryo-EM density maps). The available maps were downloaded for the study. The 128 standard test data were identified and removed from the downloaded maps. Furthermore, a few maps were removed since we were unable to locate the corresponding protein structures in the Protein Data Bank (PDB). The dataset was then split into training and validation sets.<br><br>The 500 new test datasets were downloaded after April 2023. These maps were released after April 2023 and were not present in the training and validation dataset
Data exclusions	*Describe any data exclusions. If no data were excluded from the analyses, state so OR if data were excluded, describe the exclusions and the rationale behind them, indicating whether exclusion criteria were pre-established.*
Replication	The source code for Cryo2Struct is open-source and available in the GitHub repository, accessible here: https://github.com/jianlin-cheng/Cryo2Struct. A self-contained and fully functional source code is also available in Code Ocean for fast and easy replication of the results, available here: https://codeocean.com/capsule/1820995/tree. The source codes are also uploaded into Zenodo with DOI : https://doi.org/10.5281/zenodo.11492584<br><br>Both the GitHub source code and the Code Ocean code can be used by users to reproduce the experimental results. We were able to reproduce the results when we ran Cryo2Struct multiple times for the same test data
Randomization	*Describe how samples/organisms/participants were allocated into experimental groups. If allocation was not random, describe how covariates were controlled OR if this is not relevant to your study, explain why.*
Blinding	*Describe whether the investigators were blinded to group allocation during data collection and/or analysis. If blinding was not possible, describe why OR explain why blinding was not relevant to your study.*

Reporting for specific materials, systems and methods

We require information from authors about some types of materials, experimental systems and methods used in many studies. Here, indicate whether each material, system or method listed is relevant to your study. If you are not sure if a list item applies to your research, read the appropriate section before selecting a response.

Materials & experimental systems

n/a	Involved in the study	
[x]	[ ]	Antibodies
[x]	[ ]	Eukaryotic cell lines
[x]	[ ]	Palaeontology and archaeology
[x]	[ ]	Animals and other organisms
[x]	[ ]	Clinical data
[x]	[ ]	Dual use research of concern
[x]	[ ]	Plants

Methods

n/a	Involved in the study	
[x]	[ ]	ChIP-seq
[x]	[ ]	Flow cytometry
[x]	[ ]	MRI-based neuroimaging

Plants

Seed stocks	*Report on the source of all seed stocks or other plant material used. If applicable, state the seed stock centre and catalogue number. If plant specimens were collected from the field, describe the collection location, date and sampling procedures.*
Novel plant genotypes	*Describe the methods by which all novel plant genotypes were produced. This includes those generated by transgenic approaches, gene editing, chemical/radiation-based mutagenesis and hybridization. For transgenic lines, describe the transformation method, the number of independent lines analyzed and the generation upon which experiments were performed. For gene-edited lines, describe the editor used, the endogenous sequence targeted for editing, the targeting guide RNA sequence (if applicable) and how the editor was applied.*
Authentication	*Describe any authentication procedures for each seed stock used or novel genotype generated. Describe any experiments used to assess the effect of a mutation and, where applicable, how potential secondary effects (e.g. second site T-DNA insertions, mosiacism, off-target gene editing) were examined.*
